# Lung clearance index in adults with non-cystic fibrosis bronchiectasis

**DOI:** 10.1186/1465-9921-15-59

**Published:** 2014-05-18

**Authors:** Sherif Gonem, Alys Scadding, Marcia Soares, Amisha Singapuri, Per Gustafsson, Chandra Ohri, Simon Range, Christopher E Brightling, Ian Pavord, Alex Horsley, Salman Siddiqui

**Affiliations:** 1 Institute for Lung Health, University of Leicester, Leicester, UK; 2 Department of Paediatrics, Central Hospital, Skövde, Sweden; 3 Manchester Adult Cystic Fibrosis Centre, Manchester, UK; 4 Institute of Inflammation and Repair, University of Manchester, Manchester, UK; 5 Respiratory BRU, Glenfield Hospital, Groby Road, Leicester LE3 9QP, UK

**Keywords:** Bronchiectasis, Lung clearance index, Ventilation heterogeneity

## Abstract

**Background:**

Lung clearance index (LCI) is a measure of abnormal ventilation distribution derived from the multiple breath inert gas washout (MBW) technique. We aimed to determine the clinical utility of LCI in non-CF bronchiectasis, and to assess two novel MBW parameters that distinguish between increases in LCI due to specific ventilation inequality (LCI_vent_) and increased respiratory dead space (LCI_ds_).

**Methods:**

Forty-three patients with non-CF bronchiectasis and 18 healthy control subjects underwent MBW using the sulphur hexafluoride wash-in technique, and data from 40 adults with CF were re-analysed. LCI_vent_ and LCI_ds_ were calculated using a theoretical two-compartment lung model, and represent the proportional increase in LCI above its ideal value due to specific ventilation inequality and increased respiratory dead space, respectively.

**Results:**

LCI was significantly raised in patients with non-CF bronchiectasis compared to healthy controls (9.99 versus 7.28, p < 0.01), and discriminated well between these two groups (area under receiver operating curve = 0.90, versus 0.83 for forced expiratory volume in one second [% predicted]). LCI, LCI_vent_ and LCI_ds_ were repeatable (intraclass correlation coefficient > 0.75), and correlated significantly with measures of spirometric airflow obstruction.

**Conclusion:**

LCI is repeatable, discriminatory, and is associated with spirometric airflow obstruction in patients with non-CF bronchiectasis. LCI_vent_ and LCI_ds_ are a practical and repeatable alternative to phase III slope analysis and may allow a further level of mechanistic information to be extracted from the MBW test in patients with severe ventilation heterogeneity.

## Introduction

Non-cystic fibrosis (CF) bronchiectasis is a chronic suppurative lung disease caused by a range of underlying conditions, which is increasing in prevalence [1], and which imposes a significant burden of morbidity and healthcare costs. In the United States alone, annual healthcare costs for bronchiectasis are estimated as $630 million [2]. The causes of non-CF bronchiectasis are diverse, and include autoimmune disease, primary ciliary dyskinesia, allergic bronchopulmonary aspergillosis, immune deficiency and childhood respiratory infection [3]. Regardless of the underlying cause, the pathogenesis is thought to involve a vicious cycle of bacterial colonisation, neutrophilic airway inflammation, airway damage and mucus stasis [3]. The evidence base for the treatment of non-CF bronchiectasis lags well behind that of CF, but this is expected to change in the near future as a number of non-CF bronchiectasis research registries and clinical trials are actively enrolling patients at present [4]. Such clinical trials will require robust physiological outcome measures in order to provide objective measures of improvement in lung function.

Multiple breath inert gas washout (MBW) is a technique for quantifying ventilation heterogeneity, the uneven distribution of ventilation [5]. This is an early feature of obstructive airway diseases such as asthma [6], chronic obstructive pulmonary disease [6] and cystic fibrosis (CF) [7]. A comprehensive standardisation document for the performance of inert gas washout has been recently published [8]. Lung clearance index (LCI) [9,10] is the most commonly reported MBW parameter, and is defined as the cumulative expired volume at the point where end-tidal inert gas concentration falls below 1/40th of the original concentration, divided by the functional residual capacity (FRC). LCI has been shown to be both discriminatory and repeatable in patients with CF [7], and is increasingly being used as an outcome measure in clinical trials of CF therapies [11-13]. A recent study has shown that LCI is repeatable in patients with non-CF bronchiectasis, and correlates with computed tomography bronchiectasis severity scores [14].

Although LCI has been shown to be a robust and repeatable measurement in patients with CF and non-CF bronchiectasis, it also represents a simplification of the washout process since it is essentially determined by data points at the start and end of the washout curve only. From a theoretical standpoint, LCI may be increased by two distinct mechanisms, namely (i) unequal convective ventilation between relatively large lung units subtended by proximal conducting airways (convection-dependent inhomogeneity), and (ii) increased respiratory dead space, which is thought to be underpinned by diffusion-dependent gas mixing inefficiencies (diffusion-convection-dependent inhomogeneity) [15]. The only published method for separating out these mechanisms is the analysis of phase III slopes, yielding the parameters S_cond_ (conductive ventilation heterogeneity index) and S_acin_ (acinar ventilation heterogeneity index) [16]. This technique was developed from elegant clinical and modelling studies in healthy adult subjects [17]. However, the use of these parameters is problematic in patients with the most severe ventilation heterogeneity, such as those with advanced CF lung disease [18], both from a practical standpoint (the requirement for controlled 1 L breaths) and because the modelling may not be directly applicable in those with severe ventilation heterogeneity. To overcome this, modified versions of these parameters (S_cond_* and S_acin_*) have recently been proposed for use in such patients [19]. There remains a need however for a reliable and repeatable method of extracting mechanistic information from washout curves, which has been developed for, and can be applied in, those with more severe disease.

The primary aim of this study was to determine whether or not ventilation heterogeneity is a significant feature of non-CF bronchiectasis, and whether LCI may have potential as an outcome measure in this group of patients. A further aim of the study was to extend currently available measures of ventilation heterogeneity by developing novel parameters that would distinguish between specific ventilation inequality (LCI_vent_) and increased respiratory dead space (LCI_ds_) as a cause of increased LCI. LCI_vent_ and LCI_ds_ would be expected to probe similar mechanisms of ventilation heterogeneity to S_cond_ and S_acin_, respectively, but without the potential drawbacks of phase III slope analysis, and with the advantage of being directly linked to LCI.

We hypothesised that:

i) Non-CF bronchiectasis is characterised by increased LCI, LCI_vent_ and LCI_ds_ compared to healthy control subjects.

ii) LCI is related to other measures of disease severity in CF and non-CF bronchiectasis, namely the degree of spirometric airflow obstruction and the presence or absence of chronic bacterial colonisation.

iii) LCI is repeatable in patients with non-CF bronchiectasis, and is superior to spirometry for distinguishing between patients with non-CF bronchiectasis and healthy controls.

## Methods

### Subjects

Forty-three adult patients with non-CF bronchiectasis were recruited from the respiratory out-patient clinics at Glenfield Hospital. Bronchiectasis was diagnosed by high resolution computed tomography, and all scans were reported by a Consultant Radiologist to confirm the diagnosis. Eighteen healthy non-smoking control subjects with no history of respiratory symptoms were recruited through local advertising. The study was approved by the National Research Ethics Committee (East Midlands – Leicester), and all participants gave their written informed consent. As a disease comparator group, MBW data from 40 adults with CF who took part in a previous observational study [7] were re-analysed. This study was approved by the Lothian Research and Ethics Committee and all participants gave their written informed consent.

### Clinical characterisation of bronchiectasis patients

Demographic details and a full medical history were obtained from each patient. Sputum samples were obtained for bacterial culture, and sputum culture results during the previous year were recorded to assess for chronic bacterial colonisation, defined as isolation of the same microorganism on sputum culture on at least two occasions during the previous year. Participants underwent spirometry and measurement of lung volumes using helium dilution according to American Thoracic Society/European Respiratory Society guidelines [20,21].

### Multiple breath washout test

MBW was performed in triplicate at a single visit, using the method described by Horsley *et al.*[7]. Participants wore a nose clip and breathed a known concentration (0.2%) of an inert and non-absorbed gas, sulphur hexafluoride (SF_6_), via a mouthpiece connected to an Innocor photoacoustic gas analyser (Innovision AS, Odense, Denmark), until the expired concentration in their exhaled breath reached a steady state (wash-in phase). Participants with non-CF bronchiectasis maintained a steady respiratory rate of approximately 12 breaths per minute, and a constant tidal volume of 1 L, using a real-time visual display of inspired volume as a guide. Patients with CF in the previously published cohort [7] were not generally able to follow this protocol, and washout tests were therefore performed during relaxed tidal breathing in this group. Following completion of wash-in, participants were rapidly switched to breathing room air during expiration and continued the same pattern of breathing (washout phase). Washout continued until the end-tidal concentration of expired SF_6_ fell below 1/40th of the original concentration (ie. < 0.005%) for three consecutive breaths.

### Analysis of washout curves

Washout curves were analysed using custom software written with TestPoint (Measurement Computing Corporation, Norton, Massachusetts, USA). FRC was calculated by dividing the total volume of SF_6_ expired during the washout by the difference between the SF_6_ concentrations at the beginning and end of the washout period [22]. LCI was calculated as the cumulative expired volume at the point where the end-tidal concentration of expired SF_6_ fell below 1/40th of the original concentration, divided by the FRC [9]. S_cond_/S_acin_ and S_cond_*/S_acin_* were calculated as described by Verbanck *et al.*[16,19]. The derivation of the novel parameters LCI_vent_ and LCI_ds_ is described in detail in the Additional files 1,2,3,4,5,6,7 and 8. Briefly, washout curves were fitted to a theoretical two-compartment lung model. The output parameters of the model were (i) the ratio of the specific ventilations of the two compartments, and (ii) the effective respiratory dead space. These parameters were then utilised to derive:

i. LCI_vent_ – The proportional increase in LCI above its ideal value due to specific ventilation inequality.

ii. LCI_ds_ – The proportional increase in LCI above its ideal value due to increased respiratory dead space.

Table 1 summarises the MBW parameters calculated in this study and their physiological interpretation.

**Table 1:** Multiple breath washout parameters

	**Method of calculation**	**Mechanism of ventilation heterogeneity**
LCI	Analysis of basic washout curve	Summary measure of overall ventilation heterogeneity
LCI_vent_	Two-compartment model	Specific ventilation inequality: convection-dependent
LCI_ds_	Two-compartment model	Dead space contribution: diffusion-convection-dependent
S_cond_	Phase III slope analysis	Convection-dependent
S_acin_	Phase III slope analysis	Diffusion-convection-dependent
S_cond_*	Phase III slope analysis (modified)	Convection-dependent
S_acin_*	Phase III slope analysis (modified)	Diffusion-convection-dependent

### Statistical analysis

Statistical analyses were performed using SPSS Version 20 (IBM Corporation, Somers, New York, USA) and Prism 6 (GraphPad Software Inc., La Jolla, California, USA). Between-group comparisons were performed using Student’s *T* test or one-way analysis of variance for continuous data and the Chi-squared test for proportions, with the threshold for statistical significance set at p < 0.05. Repeatability of MBW parameters was assessed using the intraclass correlation coefficient (ICC) across triplicate measurements, using a two-way mixed model. Correlations between variables were assessed using Pearson’s correlation coefficient (R). A generalised linear model was used to assess whether the relationship between LCI and spirometric airflow obstruction differed between the two disease groups. Areas under receiver operating characteristic (ROC) curves were used to assess the discriminatory ability of physiological parameters.

## Results

### Clinical characteristics

The cohort of patients with non-CF bronchiectasis comprised 19 men and 24 women with a mean (standard deviation [SD]) age of 67.4 (7.3) years. The group included 25 never smokers, 17 ex-smokers and 1 current smoker. The median (range) pack-year smoking history of the ex- and current smokers was 17.5 (1 – 140). Out of the 43 patients, a previous history of tuberculosis was elicited in 2 patients, childhood pneumonia in 14 patients and pertussis in 22 patients. Eleven patients had a history of asthma, and four had a formal diagnosis of allergic bronchopulmonary aspergillosis. Nineteen patients had symptoms of gastroesophageal reflux disease and two had inflammatory bowel disease. Twelve patients had an inflammatory arthritis and one had yellow nail syndrome. Twelve patients were chronically colonised with *Haemophilus influenzae*, three patients with *Pseudomonas aeruginosa,* two patients with *Staphylococcus aureus* and two patients with coliforms.

The CF group comprised 20 men and 20 women with a mean (SD) age of 28.7 (9.8) years. Three CF patients were ex-smokers (pack-year histories of 5, 15 and 24 years). Fifteen patients had chronic *Pseudomonas aeruginosa* colonisation as defined by Lee *et al.*[23], 29 had pancreatic insufficiency and 6 had diabetes mellitus. Nineteen patients had a severe genotype, defined as having a class I or II mutation on both chromosomes, and 16 had a mild genotype, defined as having a class III, IV or V mutation on at least one chromosome. The genotype was incomplete in 5 patients.

### Group comparisons

**Figure 1:**
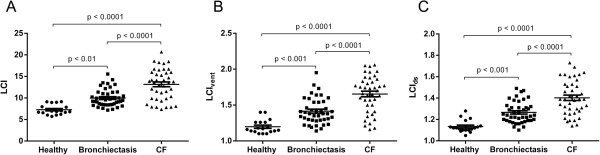
**Multiple breath washout parameters across groups.** CF = cystic fibrosis; LCI = lung clearance index; LCIvent = specific ventilation inequality component of lung clearance index; LCIds = dead space component of lung clearance index. LCI **(Panel A)**, LCIvent **(Panel B)** and LCIds **(Panel C)** are compared across groups. Error bars denote mean +/- standard error.

Table 2 shows physiological data across all three groups. Patients with bronchiectasis and CF both displayed spirometric airflow obstruction, with significantly reduced forced expiratory volume in one second/forced vital capacity (FEV_1_/FVC) ratio compared to healthy controls. LCI, LCI_vent_ and LCI_ds_ were all significantly greater in bronchiectasis patients compared to controls, and significantly greater in CF patients compared to both controls and bronchiectasis patients (see Figure 1).

**Table 2:** Demographic and physiological data across groups

	**Control subjects (n = 18)**	**CF patients (n = 40)**	**Non-CF bronchiectasis patients (n = 43)**
Age (years)^‡‡‡‡^	48.3 (3.9)	28.7 (1.5)^####^	67.4 (1.1)^####, ¥¥¥¥^
Sex (% male)	50	50	44
BMI (kg/m^2^)^‡‡‡‡^	26.8 (1.2)	22.9 (0.5)^##^	27.1 (0.7)^¥¥¥¥^
FEV_1_ (% pred.)^‡‡‡‡^	113.3 (4.8)	65.9 (3.4)^####^	82.0 (3.8)^####, ¥¥^
FVC (% pred.)^‡‡‡‡^	117.2 (5.6)	84.5 (3.0)^####^	96.1 (3.4)^##, ¥^
FEV_1_/FVC (%)^‡‡‡‡^	80.9 (1.0)	65.9 (1.8)^####^	68.4 (1.7)^####^
FRC_mbw_ (L)^‡‡^	2.52 (0.19)	1.99 (0.09)^#^	2.48 (0.10)^¥¥^
LCI^‡‡‡‡^	7.28 (0.27)	13.17 (0.56)^####^	9.99 (0.31)^##, ¥¥¥¥^
LCI_vent_^‡‡‡‡^	1.20 (0.02)	1.65 (0.04)^####^	1.42 (0.03)^###, ¥¥¥¥^
LCI_ds_^‡‡‡‡^	1.13 (0.01)	1.40 (0.03)^####^	1.27 (0.02)^###, ¥¥¥¥^
S_cond_ (L^‒1^)^‡‡‡‡^	0.033 (0.007)	0.131 (0.010)^####^	0.064 (0.007)^¥¥¥¥^
S_acin_ (L^‒1^)^‡‡‡‡^	0.118 (0.014)	0.509 (0.056)^####^	0.373 (0.036)^##^
S_cond_* (L^‒1^)^‡‡‡‡^	0.097 (0.009)	0.308 (0.034)^####^	0.107 (0.010)^¥¥¥¥^
S_acin_* (L^‒1^)^‡‡‡‡^	0.090 (0.012)	0.446 (0.054)^####^	0.355 (0.037)^##^

CF = cystic fibrosis; BMI = body mass index; FEV_1_ = forced expiratory volume in one second; FVC = forced vital capacity; FRC_mbw_ = functional residual capacity from multiple breath washout; LCI = lung clearance index; LCI_vent_ = specific ventilation inequality component of lung clearance index; LCI_ds_ = dead space component of lung clearance index. Data expressed as mean (standard error) or percentages. Groups compared using one-way analysis of variance with Bonferroni correction for multiple comparisons for parametric data, and the Chi-squared test for proportions. Significant differences across groups denoted ^‡‡^(p < 0.01) or ^‡‡‡‡^(p < 0.0001). Significant differences compared to control group denoted ^#^(p < 0.05), ^##^(p < 0.01), ^###^(p < 0.001) or ^####^(p < 0.0001). Significant differences between bronchiectasis and CF groups denoted ^¥^(p < 0.05), ^¥¥^(p < 0.01) or ^¥¥¥¥^(p < 0.0001).

### Correlations between spirometric airflow obstruction and lung clearance index

**Figure 2:**
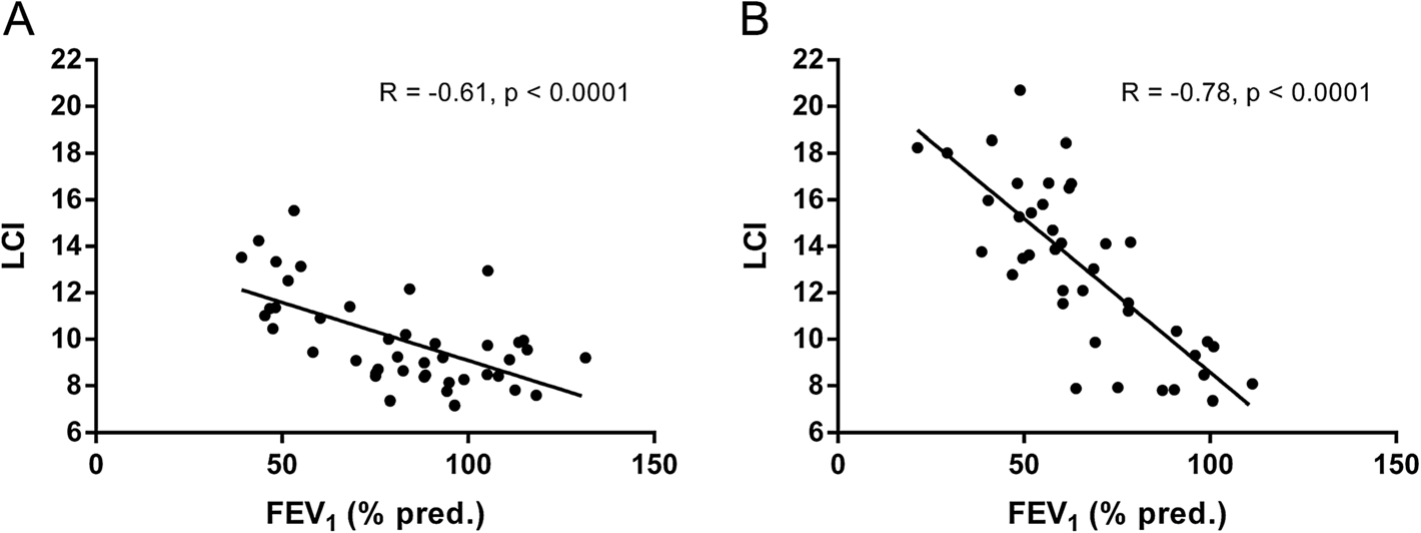
**Correlations between lung clearance index and FEV**_**1**_**(% predicted).** Correlations are shown for patients with non-cystic fibrosis bronchiectasis **(Panel A)** and cystic fibrosis **(Panel B)**. LCI = lung clearance index; FEV_1_ = forced expiratory volume in one second. Best-fit linear regression lines are shown, together with Pearson correlation coefficients.

Figure 2 shows correlations between the FEV_1_ (% pred.) and LCI in patients with bronchiectasis and patients with CF. In both cases, there was a highly significant (p < 0.0001) negative correlation between FEV_1_ (% pred.) and LCI. However, the slope of the relationship between the two variables differed significantly between the groups. Patients with CF had a 0.13 unit increase in LCI for every 1 percentage point reduction in FEV_1_ (% pred.), whereas patients with bronchiectasis had a 0.05 unit increase in LCI for every 1 percentage point reduction in FEV_1_ (% pred.) (p < 0.0001). LCI_vent_ and LCI_ds_ correlated highly significantly with FEV_1_ (% pred.) in both patients with non-CF bronchiectasis (R = -0.63 for LCI_vent_, R = -0.60 for LCI_ds_, p < 0.0001 for both analyses) and patients with CF (R = -0.78 for LCI_vent_, R = -0.76 for LCI_ds_, p < 0.0001 for both analyses). There were significant correlations between LCI_vent_ and LCI_ds_ in both patient groups (R = 0.80, p < 0.0001 for non-CF bronchiectasis; R = 0.89, p < 0.0001 for CF).

### Multiple breath washout parameters and chronic bacterial colonisation

Table 3 shows MBW and spirometric indices in patients with CF in the presence and absence of chronic *P. aeruginosa* colonisation, and in patients with non-CF bronchiectasis in the presence and absence of chronic bacterial colonisation. LCI_ds_ was significantly raised in CF patients with chronic *P. aeruginosa* colonisation compared to those without chronic colonisation (1.49 vs 1.34, p = 0.004).

**Table 3:** Physiological parameters in patients with and without chronic bacterial colonisation

	**Non-cystic fibrosis bronchiectasis**		**Cystic fibrosis**	
	**No chronic colonisation**	**Chronic colonisation**	**No chronic PsA colonisation**	**Chronic PsA colonisation**
	**(n = 26)**	**(n = 17)**	**(n = 25)**	**(n = 15)**
FEV_1_ (% pred.)	86.1 (5.3)	75.6 (4.9)	69.7 (4.5)	59.6 (4.5)
FVC (% pred.)	101.7 (4.6)	87.5 (4.1)^#^	86.9 (3.6)	80.6 (5.5)
FEV_1_/FVC (%)	68.6 (2.4)	68.1 (2.4)	67.1 (2.5)	63.9 (2.5)
TLC (% pred.)	95.3 (3.1)	93.9 (4.0)	-	-
LCI	10.02 (0.36)	9.95 (0.57)	12.29 (0.72)	14.44 (0.85)
LCI_vent_	1.42 (0.03)	1.41 (0.05)	1.59 (0.05)	1.75 (0.05)
LCI_ds_	1.27 (0.02)	1.26 (0.03)	1.34 (0.03)	1.49 (0.04)^##^
S_cond_ (L^‒1^)	0.058 (0.010)	0.072 (0.010)	0.122 (0.010)	0.132 (0.018)
S_acin_ (L^‒1^)	0.429 (0.053)	0.288 (0.038)	0.438 (0.070)	0.611 (0.093)
S_cond_* (L^‒1^)	0.102 (0.014)	0.115 (0.016)	0.284 (0.034)	0.294 (0.051)
S_acin_* (L^‒1^)	0.412 (0.053)	0.268 (0.041)	0.376 (0.068)	0.553 (0.092)

PsA = *Pseudomonas aeruginosa*; FEV_1_ = forced expiratory volume in one second; FVC = forced vital capacity; TLC = total lung capacity; LCI = lung clearance index; LCI_vent_ = specific ventilation inequality component of lung clearance index; LCI_ds_ = dead space component of lung clearance index. Data expressed as mean (standard error). Colonised and non-colonised groups within each disease cohort compared using Student’s *T* test. Significant differences between groups denoted ^#^(p < 0.05) or ^##^(p < 0.01).

### Within-visit repeatability and discriminatory ability

**Figure 3:**
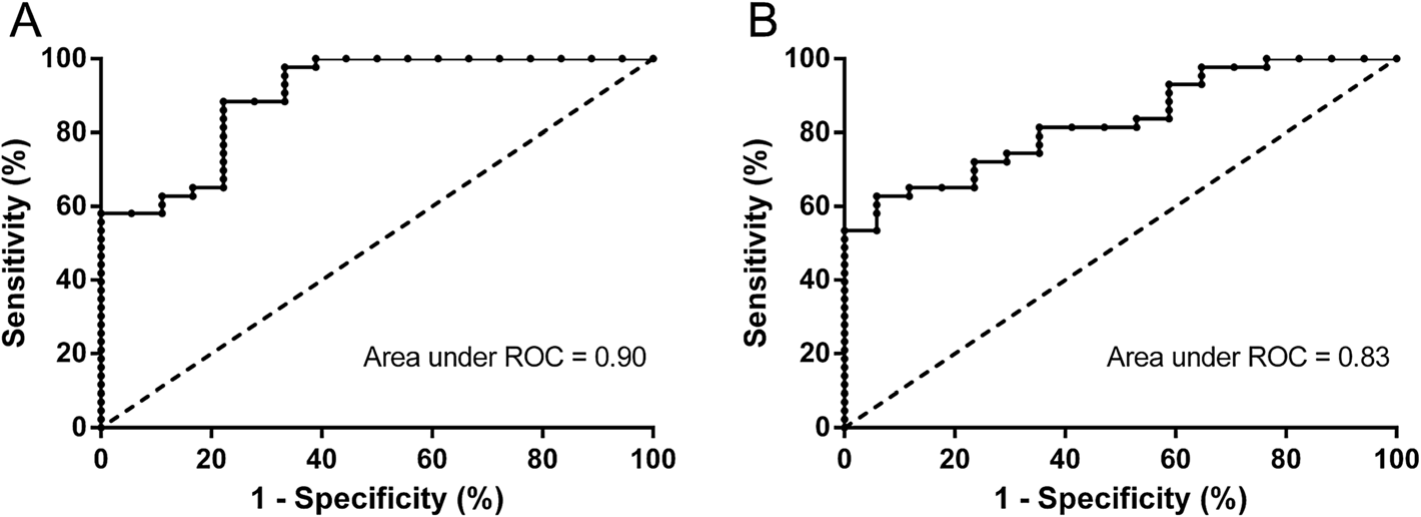
**Receiver operating characteristic curves of lung clearance index and FEV**_**1**_**(% pred.) for distinguishing between control subjects and bronchiectasis patients.** Receiver operating characteristic (ROC) curves are shown for lung clearance index (LCI) **(Panel A)** and forced expiratory volume in one second percent predicted (FEV_1_ [% pred.]) **(Panel B)**. Areas under ROC curves are 0.90 for LCI and 0.83 for FEV_1_ (% pred.).

Table 4 shows the repeatability of MBW parameters in patients with bronchiectasis and CF. Intraclass correlation coefficients exceeded 0.75 for LCI, LCI_vent_ and LCI_ds_ in both disease groups. S_acin_ and S_acin_* displayed moderate or good repeatability, but S_cond_ and S_cond_* were poorly repeatable in both disease groups. Figure 3 shows ROC curves illustrating the discriminatory ability of LCI and FEV_1_ (% pred.) for distinguishing between healthy controls and patients with non-CF bronchiectasis. The area under the ROC curve was 0.90 for LCI and 0.83 for FEV_1_ (% pred.). Areas under the ROC curve for LCI_vent_ and LCI_ds_ were 0.88 and 0.89, respectively.

**Table 4:** Within-visit repeatability of multiple breath washout parameters

	**ICC in non-CF bronchiectasis**	**ICC in CF**
LCI	0.86	0.93
LCI_vent_	0.91	0.89
LCI_ds_	0.79	0.88
S_cond_	0.15	0.19
S_acin_	0.63	0.88
S_cond_*	0.10	0.41
S_acin_*	0.65	0.90

ICC = intraclass correlation coefficient; SD = standard deviation; CF = cystic fibrosis; LCI = lung clearance index; LCI_vent_ = specific ventilation inequality component of lung clearance index; LCI_ds_ = dead space component of lung clearance index.

**Figure 4:**
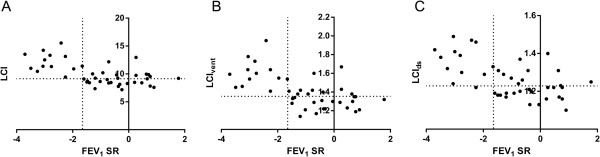
**Scatterplots of forced expiratory volume in one second standardised residuals against multiple breath washout parameters.** LCI = lung clearance index; LCIvent = specific ventilation inequality component of lung clearance index; LCIds = dead space component of lung clearance index; FEV1 = forced expiratory volume in one second; SR = standardised residuals. FEV1 SR are plotted against LCI **(Panel A)**, LCIvent **(Panel B)** and LCIds **(Panel C)**. Dotted lines denote the lower limit of normal for FEV1 and upper limits of normal for LCI, LCIvent and LCIds.

Figure 4 shows graphs of FEV_1_ standardised residuals against LCI, LCI_vent_ and LCI_ds_ in patients with non-CF bronchiectasis. The lower limit of normal for FEV_1_ was defined as 1.645 standard deviations below the predicted value, while the upper limits of normal for LCI, LCI_vent_ and LCI_ds_ were defined as the mean + 1.645 standard deviations in the healthy control group. Thirty out of 43 patients had an FEV_1_ within the normal range, and of these, LCI, LCI_vent_ and LCI_ds_ were high in 12, 10 and 10 patients, respectively. Conversely, there were no patients who had an FEV_1_ below the normal range who did not also have a raised LCI and LCI_vent_, and only one patient who had an FEV_1_ below the normal range with a normal LCI_ds_.

## Discussion

We have shown that LCI, and the novel parameters LCI_vent_ and LCI_ds_, are significantly raised in patients with non-CF bronchiectasis compared to controls, and that these parameters correlate strongly with spirometric markers of airflow obstruction. LCI, LCI_vent_ and LCI_ds_ display good within-visit repeatability in patients with non-CF bronchiectasis, and superior discriminatory ability for distinguishing bronchiectasis patients from controls compared to FEV_1_. Moreover, these parameters are abnormally raised in a significant proportion of non-CF bronchiectasis patients with a normal FEV_1_. These findings suggest that MBW parameters may have potential as markers of disease severity in patients with non-CF bronchiectasis, and may be indicators of incipient airflow obstruction, although longitudinal studies are required to test this hypothesis. Further studies are also required to determine the between-visit variability and minimal clinically important difference of MBW parameters in patients with non-CF bronchiectasis, as well as their responsiveness to therapeutic interventions.

A major aim of this study was to develop novel markers of ventilation heterogeneity that would distinguish between the two possible mechanisms of increased LCI, namely specific ventilation inequality and increased respiratory dead space. Previous studies have used measures of the curvilinearity of the washout curve as markers of specific ventilation inequality, but these methods did not provide a formal estimate of the respiratory dead space component [15,19]. Although in healthy subjects it is thought that specific ventilation inequality is the only mechanism of ventilation heterogeneity operative at the level of the proximal conducting airways, the situation is disease is far more complex. Depending on the extent of airway damage and obstruction, diffusion may not be neatly compartmentalised to the distal airways. An advantage of the current method is that it does not pre-suppose an anatomical location for the observed abnormalities in ventilation heterogeneity, but concentrates on the underlying mechanisms. This is particularly relevant when dealing with those with more severe airflow obstruction and ventilation heterogeneity. Furthermore, since the proximal and distal airways are not independent of each other, and form a complex interacting network [24], it is also unsurprising that we noted a correlation between LCI_vent_ and LCI_ds_ in both patient groups. LCI_vent_ and LCI_ds_ may however allow subtle distinctions to be made in terms of mechanisms of disease in airway diseases such as CF and non-CF bronchiectasis. Indeed, we observed that CF patients with chronic *P. aeruginosa* colonisation had increased LCI_ds_ compared to those who did not, whereas LCI_vent_ did not differ significantly between the groups. This extends the findings of Belessis *et al.*[25], who observed that LCI was higher in children with CF who had *P. aeruginosa* colonisation compared to those who did not. Our results suggest that this increase in LCI may be driven predominantly by an increased respiratory dead space. Interestingly, neither MBW parameters nor FEV_1_ (% pred.) differed significantly between non-CF bronchiectasis patients with and without chronic bacterial colonisation. Chronic colonisation in our cohort was mainly with *H. influenzae* rather than *P. aeruginosa*, and our data therefore concord with previous observations that *H. influenzae*, unlike *P. aeruginosa*, is not associated with faster lung function decline in non-CF bronchiectasis [26]. The reduced FVC (% pred.) we observed in non-CF bronchiectasis patients with chronic colonisation was not associated with an abnormally low TLC (% pred.), and therefore did not represent a true restrictive deficit.

Previous attempts to apply phase III slope analysis in CF were less successful than in reports from other disease groups, because of both poor repeatability and reliance of the original method on a strict 1 L breathing protocol [18]. Although it relies on a relaxed and repeatable breathing pattern, the current method does not require strict breath volume control, something patients often find harder to maintain than well-trained volunteers. In addition, LCI_vent_ and LCI_ds_ showed superior repeatability to phase III slope parameters, in particular to S_cond_ and S_cond_*. This is an important attribute if these measures are to be used to assess change over time, or in response to therapeutic intervention.

A potential limitation of our study was that the disease groups were not matched for age with the control group. This was to a certain extent unavoidable, since patients with non-CF bronchiectasis are in general older than those with CF, and we therefore chose our control group to be approximately intermediate in age between the two disease groups. However, recently published regression equations [27] indicate that the effects of this on our results were likely to be modest – in particular, LCI is expected to increase by 0.0223 units per year, so the 19-year difference in mean age between patients with bronchiectasis and healthy controls would be predicted to cause a relatively small 0.43 difference in LCI between the groups. Furthermore, the upper limit of normal of LCI derived from our healthy control data was slightly higher than that reported in previous studies using the same methodology [7], a difference that may be explained by the older age of our healthy cohort. Further studies are required to derive age- and sex-dependent normative ranges for LCI using the SF_6_ wash-in method, as have been published for nitrogen washout [27].

In conclusion, we have shown that LCI, a marker of impaired gas mixing derived from the MBW test, is significantly raised in patients with non-CF bronchiectasis, and that this elevation correlates with spirometric airflow obstruction. LCI is repeatable and discriminatory in patients with non-CF bronchiectasis, and future studies are now required to assess the prognostic significance of a raised LCI in this patient group, as well as the potential utility of this marker as an outcome measure in interventional trials. The novel parameters LCI_vent_ and LCI_ds_ are a practical and repeatable alternative to phase III slope analysis and may allow a further level of mechanistic information to be obtained from the MBW test without any additional demand on the patient. They should be reported in conjunction with LCI in future observational and interventional studies that incorporate the MBW technique.

## Competing interests

CEB has received grant funding from Roche-Genentech, Novartis, AZ-MedImmune, Chiesi and GSK; consultancy fees from Hoffmann-La Roche, AZ, GSK, Novartis, Chiesi and Merck; and funding for travel to scientific meetings from Boehringer Ingelheim. SS has received research grants from Chiesi to study small airway microstructure and has received lecturing fees from Chiesi and GSK and consultancy fees from Teva. SG has received funding for travel to scientific meetings from GSK and Chiesi. AH has received a grant from the National Institute for Health Research to investigate lung clearance index. Part of this involves a collaboration with Innovision ApS, the manufacturers of the Innocor gas analyser used in the study. IP has received speaker's fees, honoraria for attending advisory boards and travel expenses from GSK, AZ, Boehringer Ingelheim, Napp, Novartis, Aerocrine and Boston Scientific. A Scadding, MS, A Singapuri, PG, SR and CO have no conflicts of interest to declare.

## Authors’ contributions

SG – Analysed washout curves, derived novel indices, performed statistical analysis of the data and wrote the manuscript. A Scadding – Recruited patients, characterised them clinically, and performed washout tests. MS – Performed washout tests. A Singapuri – Recruited patients and performed washout tests. PG – Assisted with setting up the inert gas washout system, and critically appraised the manuscript. CO – Recruited patients and critically appraised the manuscript. SR – Recruited patients and critically appraised the manuscript. CEB – Involved in study conception and design, and critically appraised the manuscript. IP – Involved in study conception and design, and critically appraised the manuscript. AH – Involved in study conception and design, and supervised the writing of the manuscript. SS – Involved in study conception and design, and supervised the writing of the manuscript. All authors read and approved the final manuscript.

## Authors’ information

Alex Horsley and Salman Siddiqui are co-senior authors.

## Supplementary Material

Additional file 1 **Derivation of the indices LCIvent and LCIds (Additional files **2, 3, 4, 5, 6, 7 and 8). Click here for file

Additional file 2: Figure E1 Washout curve from a healthy subject. C_et_ = end-tidal SF_6_ concentration; TO = turnover number. Click here for file

Additional file 3: Figure E2 Simulated washout curves with varying specific ventilation inequality and effective respiratory dead space. C_et_ = end-tidal SF_6_ concentration; TO = turnover number. Panel A shows simulated washout curves with low (continuous line), intermediate (dashed line) and high (dotted line) levels of specific ventilation inequality. Panel B shows simulated washout curves with small (continuous line), intermediate (dashed line) and large (dotted line) effective respiratory dead space. Click here for file

Additional file 4: Figure E3 One-compartment lung model. Vd_anat_ = anatomical dead space; Vt = tidal volume; Va = alveolar volume; FRC = functional residual capacity. Click here for file

Additional file 5: Figure E4 Exponential decay curves. Single exponential decay curves of the form y = 0.2 × e^‒kx^ are shown with rate constants (k) of 0.2 (dotted line), 0.6 (dashed line) and 1.8 (continuous line). Click here for file

Additional file 6: Figure E5 Washout curves from a healthy subject and a patient with cystic fibrosis fitted to a one-phase exponential decay model. C_et (norm)_ = normalised end-tidal SF_6_ concentration; TO = turnover number. Panels A and B show washout curves from a healthy subject and a patient with cystic fibrosis, respectively, fitted to a one-phase exponential decay model. Click here for file

Additional file 7: Figure E6 Washout curves from a healthy subject and a patient with cystic fibrosis fitted to a two-phase exponential decay model. C_et (norm)_ = normalised end-tidal SF_6_ concentration; TO = turnover number. Panels A and B show washout curves from a healthy subject and a patient with cystic fibrosis, respectively, fitted to a two-phase exponential decay model. Goodness of fit (R^2^) = 0.9973 for healthy subject and 0.9775 for cystic fibrosis patient. Click here for file

Additional file 8: Figure E7 Two-compartment lung model. V_slow_ = volume of under-ventilated (slow) lung unit; V_fast_ = volume of over-ventilated (fast) lung unit; Vt = tidal volume; c = proportion of tidal volume reaching fast lung unit. Click here for file
