# Supplementary material for: Lung clearance index in adults with non-cystic fibrosis bronchiectasis
Source: Respir Res. 2014 May 18;15(1):59. doi: 10.1186/1465-9921-15-59 (PMC4035904; doi:10.1186/1465-9921-15-59)
Supplement: Additional file 1 — Derivation of the indices LCIvent and LCIds (Additional files 2, 3, 4, 5, 6, 7 and 8). [file 1465-9921-15-59-S1.rtf]

+Lung clearance index in adults with non-cystic fibrosis bronchiectasis

1Sherif Gonem, 1Alys Scadding, 1Marcia Soares, 1Amisha Singapuri, 2Per Gustafsson, 1Chandra Ohri, 1Simon Range, 1Christopher E Brightling, 1Ian Pavord, 3,4Alex Horsley*, 1Salman Siddiqui*


1Institute for Lung Health, University of Leicester, Leicester, United Kingdom
2Department of Paediatrics, Central Hospital, Skövde, Sweden
3Manchester Adult Cystic Fibrosis Centre, Manchester, United Kingdom
4Institute of Inflammation and Repair, University of Manchester, Manchester, United Kingdom
* Co senior authors


Supplementary material


Abbreviations

Cet		End-expiratory inert gas concentration
CF		Cystic fibrosis
DSeq		Equipment dead space
FRC		Functional residual capacity
LCI		Lung clearance index
LCIds		Dead space component of lung clearance index
LCIideal		Ideal lung clearance index
LCIvent		Specific ventilation inequality component of lung clearance index
MBW		Multiple breath inert gas washout
SVR		Specific ventilation ratio
TO		Turnover
Vdanat		Anatomical dead space
Vdresp		Effective respiratory dead space
VH		Ventilation heterogeneity
Vt		Tidal volume


Appendix:		Derivation of the indices LCIvent and LCIds

1	Background
Multiple breath inert gas washout (MBW) is a technique for quantifying ventilation heterogeneity (VH), the uneven distribution of ventilation, through analysis of the efficiency and pattern with which an inert tracer gas is washed out of the lungs during tidal breathing. The washout phase, from which diagnostic information is obtained, may be preceded by a wash-in phase, in which a non-resident inert tracer gas, such as 0.2% sulphur hexafluoride (SF6), is breathed until equilibrium is reached, or alternatively, resident nitrogen in the lungs may be used as the inert tracer gas. In the former case, the washout is performed by switching the subject to breathing room air, which washes out the non-resident tracer gas, and in the latter case, resident lung nitrogen is washed out by switching the subject to breathing 100% oxygen. In either case, an accurate fast-responding gas analyser is required to measure the concentration of the inert gas in expired air, while respiratory flows are measured using a pneumotachometer connected to the subject's mouthpiece. For the purposes of this appendix, it will be assumed that washouts are performed using the non-resident inert tracer gas 0.2% SF6. Following completion of a washout test, the functional residual capacity (FRC) is calculated by dividing the total volume of SF6 expired during the washout by the difference between the SF6 concentrations at the beginning and end of the washout period [1]. We calculated the anatomical dead space (Vdanat) using the method of Langley [2].

Figure E1 shows a typical washout curve in a healthy subject. Each symbol represents the end-expiratory SF6 concentration (Cet) of a single breath of the washout. As the washout proceeds, Cet decays in a roughly exponential manner until it reaches 1/40th of the initial SF6 concentration (ie. 0.005%), at which point the washout experiment is by convention stopped. The units of the x-axis are not breath number but 'turnover number' (TO), where TO is the cumulative expired volume (minus equipment dead space [DSeq]) measured in multiples of the functional residual capacity (FRC). The TO unit is used since it corrects for variations in tidal volume and FRC both within and between subjects. The most commonly reported MBW parameter is the lung clearance index (LCI), which is the number of lung turnovers taken to wash out the inert gas to 1/40th of its initial concentration. Two-compartment model simulations of gas mixing have shown that LCI may be increased by (i) unequal convective ventilation between relatively large lung units subtended by proximal conducting airways (specific ventilation inequality), or (ii) increased respiratory dead space [3]. Respiratory dead space is contributed to by Vdanat, but it is believed that inhomogeneities occurring more peripherally in the airway tree may mimic a dead space effect, thus increasing the effective respiratory dead space (Vdresp) [3]. Figure E2 shows simulated washout curves demonstrating the qualitative effect of increasing specific ventilation inequality (Panel A) or Vdresp (Panel B). Increasing specific ventilation inequality results in a change in the shape of the washout curve such that there is a rapid initial phase followed by a slow terminal phase, whereas increasing Vdresp results in a prolongation of the washout curve but without altering its shape. In both cases, the result is an increase in LCI.


2	One-compartment lung model
In order to simulate washout curves incorporating specific ventilation inequality between lung units, it is clear that an anatomical model with at least two compartments will be required. However, before introducing the two-compartment model, it is useful to briefly describe the even simpler one-compartment model of the lung. Consider the washout of a single uniformly ventilated alveolar compartment subtended by a conducting airway, as illustrated in Figure E3. The FRC is the total volume of the alveolar compartment and the conducting airway at end expiration, while the anatomical dead space (Vdanat) is the volume of the conducting airway alone, which is considered to be fixed.

Let	FRC = functional residual capacity
	Vdanat = anatomical dead space
	Vt = tidal volume

With each successive inspiration, the SF6 concentration in the alveolar compartment will be diluted by a fixed ratio. This dilution ratio was derived by Fowler et al. [4], and is equal to:


Therefore, the SF6 concentration in the alveolar compartment following the nth inspiration will be equal to the initial SF6 concentration (0.2%) multiplied by the dilution ratio raised to the power of n:


This is an exponential decay curve, of which the standard form is:


Where		a = y-intercept of the curve
		k = rate constant
 		e = the base of natural logarithms ≈ 2.718

Assuming that the initial SF6 concentration is 0.2%, the y-intercept parameter will be set at 0.2, and thus the only parameter that may vary is the rate constant k. The x-axis units may be breath number or TO number, but TO is preferred since it smoothes irregularities in the washout curve caused by variations in tidal volume. Figure E4 shows example exponential decay curves with different rate constants, illustrating that a higher rate constant results in a more rapid decay.

We used the curve-fitting software Prism 6 (GraphPad Software Inc., La Jolla, California, USA) to fit experimental washout curves (normalised to an initial SF6 concentration of 0.2%) to the above model, which is also known as a one-phase decay model. This was performed using non-linear regression with a weighting of 1/y2 in order to minimise the sum of relative, rather than absolute, squared residuals. This was in order to ensure that data points near the start of the washout curve (where absolute Cet values were higher) did not disproportionately impact upon the model fit. Figure E5 shows example washout data from a healthy control subject and a patient with cystic fibrosis (CF) fitted to the one-phase decay model. This shows that in healthy subjects, the one-phase decay model fits experimental data relatively well, whereas in patients with severe VH, the model fit is poor. Therefore, we may conclude that the one-phase decay model does not contain sufficient degrees of freedom to fit the full range of observed washout curves.


3	Two-compartment lung model
In order to simulate the washout of a two-compartment lung model we require a two-phase exponential decay curve, which has the following general form:


Where		j = fast rate constant
		k = slow rate constant
		a = weighting of fast rate constant
		b = weighting of slow rate constant

The y-intercept of this curve is equal to a + b. Since in our case this is set at 0.2 (the initial SF6 concentration), we can re-write the above equation as:


Where		c = proportionate weighting of fast-decaying component, and 0 > c > 1

This curve therefore has three variable parameters (or degrees of freedom), namely the fast rate constant (j), the slow rate constant (k) and the proportionate weighting of the fast-decaying component (c). Figure E6 shows washout curves from a healthy subject and a patient with severe CF fitted to a two-phase exponential decay curve, with an excellent model fit in both cases. 

A natural anatomical model that would be expected to behave according to the equation above is illustrated in Figure E7. It comprises two lung units in parallel, each consisting of an alveolar compartment subtended by a conducting airway. The constants j and k correspond to the rate constants for the washout of the over-ventilated (fast) and under-ventilated (slow) lung units respectively. The SF6 concentration of the expired gas from this system is equal to the weighted mean of the SF6 concentrations in each of the two alveolar compartments at end-inspiration, where the weighting is determined by the proportion of ventilation reaching each lung unit. Thus, the anatomical interpretation of the constant c, defined above, is the proportion of the tidal volume reaching the fast lung unit.

We now derive two anatomical parameters from our two-compartment model, one of which reflects specific ventilation inequality between the lung units, and the second of which reflects the effective respiratory dead space (Vdresp). A natural measure of specific ventilation inequality is the ratio of the specific ventilation of the slow lung unit to that of the fast lung unit, which we refer to as the specific ventilation ratio (SVR). 


Where		Vslow = volume of slow lung unit
	W = Vslow/FRC


As stated above, normalised Cet against TO curves can be accurately modelled by the following equation:


The subsequent analysis is simplified if this equation is expressed in terms of breath number (n) rather than TO. This change of scale is achieved by multiplying the rate constants j and k by a constant term to yield the new equation:


Where r = j  (Vt – DSeq)/FRC, and s = k  (Vt – DSeq)/FRC

Following the nth inspiration, the SF6 concentration is equal to  in the fast compartment and  in the slow compartment. Using the formula for the dilution ratio in a single compartment [4], and assuming that both tidal volume and respiratory dead space are distributed between the over-ventilated and under-ventilated lung units in a ratio of c to (1 ‒ c), we may write the following equations:


Let   and  

Then:


		
By solving these two equations in the two unknowns W and Vdresp the following formulae are derived:


Substituting:


We obtain the following formula:


4	Calculation of the indices LCIvent and LCIds
We now utilise the values of c, Vdresp and SVR derived above to determine:
i)	LCIideal – The expected value of LCI assuming no specific ventilation inequality, and no additional respiratory dead space over and above Vdanat.
ii) 	LCIvent – The proportional increase in LCI over and above LCIideal, taking into account specific ventilation inequality but assuming no additional respiratory dead space.
iii)	LCIds – The proportional increase in LCI over and above LCIideal, taking into account additional respiratory dead space, but assuming no specific ventilation inequality.

LCIideal and LCIds are calculated using the following formulae, which are based upon the dilution ratio in a single compartment, as described by Fowler et al. [4]:


In order to determine LCIvent, we utilise the values of c and SVR derived above, but set Vdresp to equal Vdanat. We then reverse the algebraic steps described in Section 3 in order to arrive at two new rate constants j' and k'. The values of c, j' and k' are then plugged into our original equation for the washout of a two-compartment model, with Cet on the y-axis and TO number on the x-axis:


Setting y = 0.005 (1/40th of the original inert gas concentration of 0.2) yields the equation:


This equation may be solved numerically to any desired degree of accuracy in the unknown x. The solution is then divided by LCIideal to yield LCIvent.


5	Step-by-step instructions for calculating LCIvent and LCIds
This section summarises the calculation of LCIvent and LCIds in a step-by-step fashion using Prism 6 (GraphPad Software Inc., La Jolla, California, USA):

Step 1
Choose an X-Y table format and enter the data with TO number on the x-axis and Cet on the y-axis. The initial SF6 concentration (ie. the SF6 concentration of expired air at the end of the wash-in phase) should be entered against a TO number of zero, and is thus the y-intercept of the curve. If a number of washout curves are to be analysed, it is convenient to normalise Cet values to a consistent initial SF6 concentration such as 0.2% so that the y-intercept of the curve is always the same. For instance, if a given washout curve has an initial SF6 concentration of 0.198%, the curve is normalised to a starting value of 0.2% by multiplying each Cet value by 0.2/0.198.

Step 2
Choose the analysis method 'non-linear regression' and fit to the equation 'two phase decay'. In the Constrain tab, set:

Y0 – Constant equal to 0.2
Plateau – Constant equal to 0
PercentFast – Must be between zero and 100.0
KFast – No constraint
KSlow – Must be greater than 0
KFast must be greater than 1.0 times KSlow

In the Weights tab, choose: Weight by 1/y2

Press 'OK' to perform the analysis. This should produce values for PercentFast, KFast and KSlow.

Step 3
Set:


Step 4
Set:


Then:


Step 5
LCIideal and LCIds are given by the following formulae:


To calculate LCIvent, utilise the values of c and SVR derived in step 4, and perform the following algebraic steps:


Using the values of c, j' and k' derived above, solve the following equation numerically in the unknown x:


Divide the value of x obtained by LCIideal to yield LCIvent.


References

1)	Darling RC, Cournand A, Richards DW. Studies on the intrapulmonary mixture of gases III. An open circuit method for measuring residual air. J Clin Invest. 1940; 19: 609-18.

2)	Langley F, Evan P, Duroux P, Nicolas RL, Cumming G. Ventilatory consequences of unilateral pulmonary artery occlusion. Coll Inst Natl Santé Rech Méd. 1975; 51: 209-12.

3)	Verbanck S, Paiva M, Schuermans D, Hanon S, Vincken W, Van Muylem A. Relationships between the lung clearance index and conductive and acinar ventilation heterogeneity. J Appl Physiol. 2012; 112: 782-90.

4)	Fowler WS, Cornish ER, Kety SS. Lung function studies VIII. Analysis of alveolar ventilation by pulmonary N2 clearance curves. J Clin Invest. 1952; 31(3): 40
