# Supplementary figures and images for: Lung clearance index in adults with non-cystic fibrosis bronchiectasis
Source: Respir Res. 2014 May 18;15(1):59. doi: 10.1186/1465-9921-15-59 (PMC4035904; doi:10.1186/1465-9921-15-59)

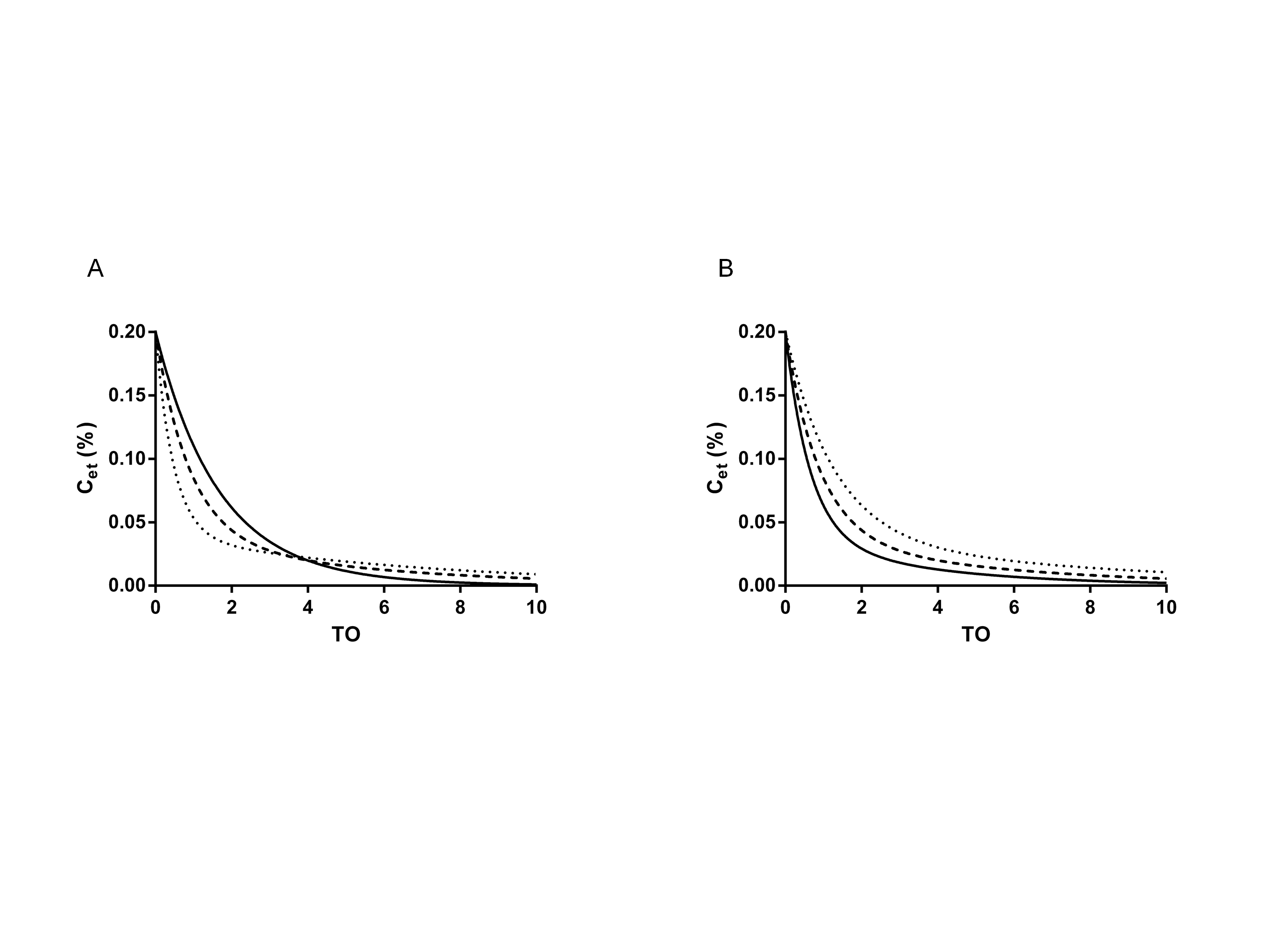

Supplement: Additional file 3: Figure E2 — Simulated washout curves with varying specific ventilation inequality and effective respiratory dead space. Cet = end-tidal SF6 concentration; TO = turnover number. Panel A shows simulated washout curves with low (continuous line), intermediate (dashed line) and high (dotted line) levels of specific ventilation inequality. Panel B shows simulated washout curves with small (continuous line), intermediate (dashed line) and large (dotted line) effective respiratory dead space. [file 1465-9921-15-59-S3.tiff]

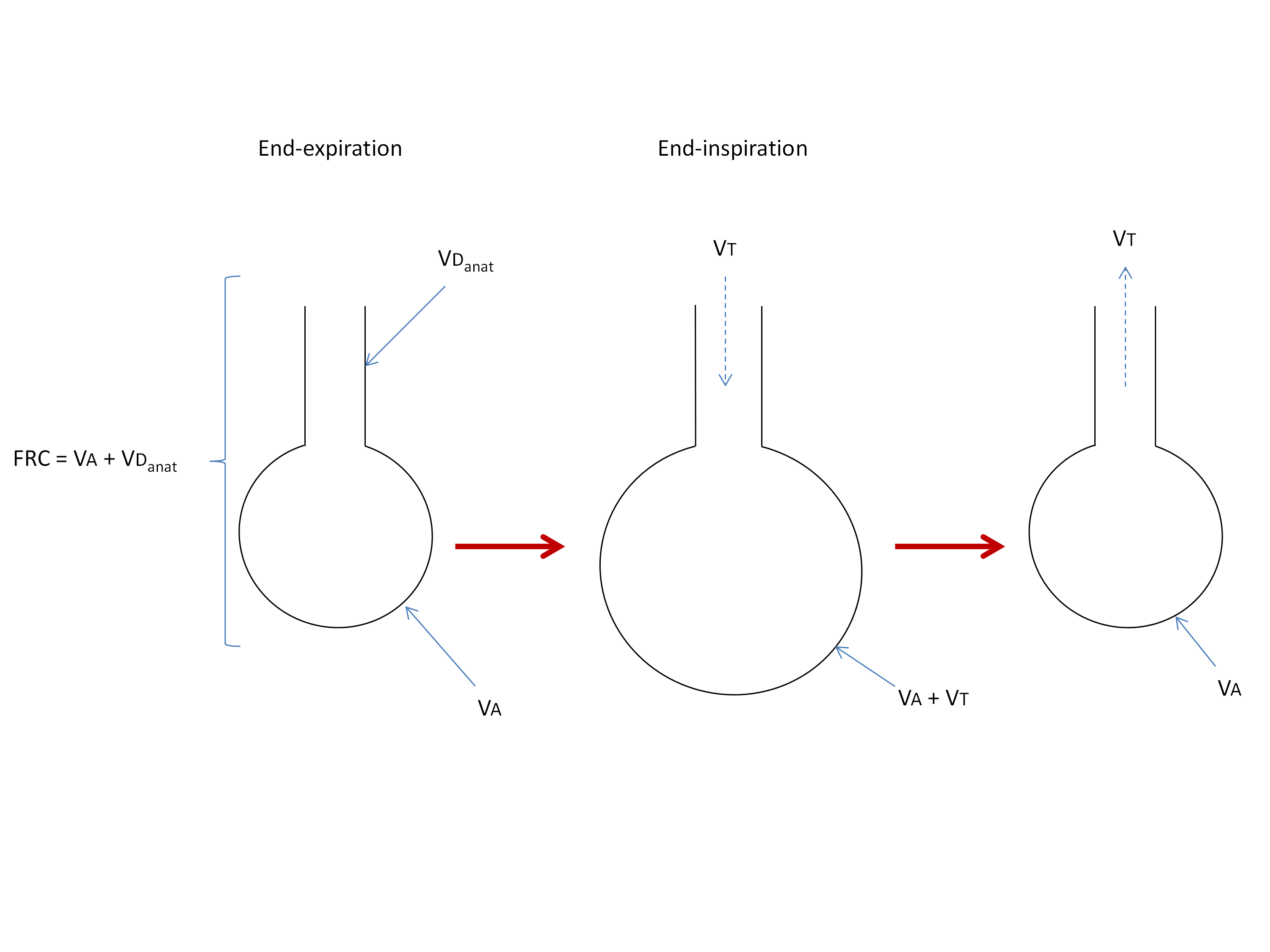

Supplement: Additional file 4: Figure E3 — One-compartment lung model. Vdanat = anatomical dead space; Vt = tidal volume; Va = alveolar volume; FRC = functional residual capacity. [file 1465-9921-15-59-S4.tiff]

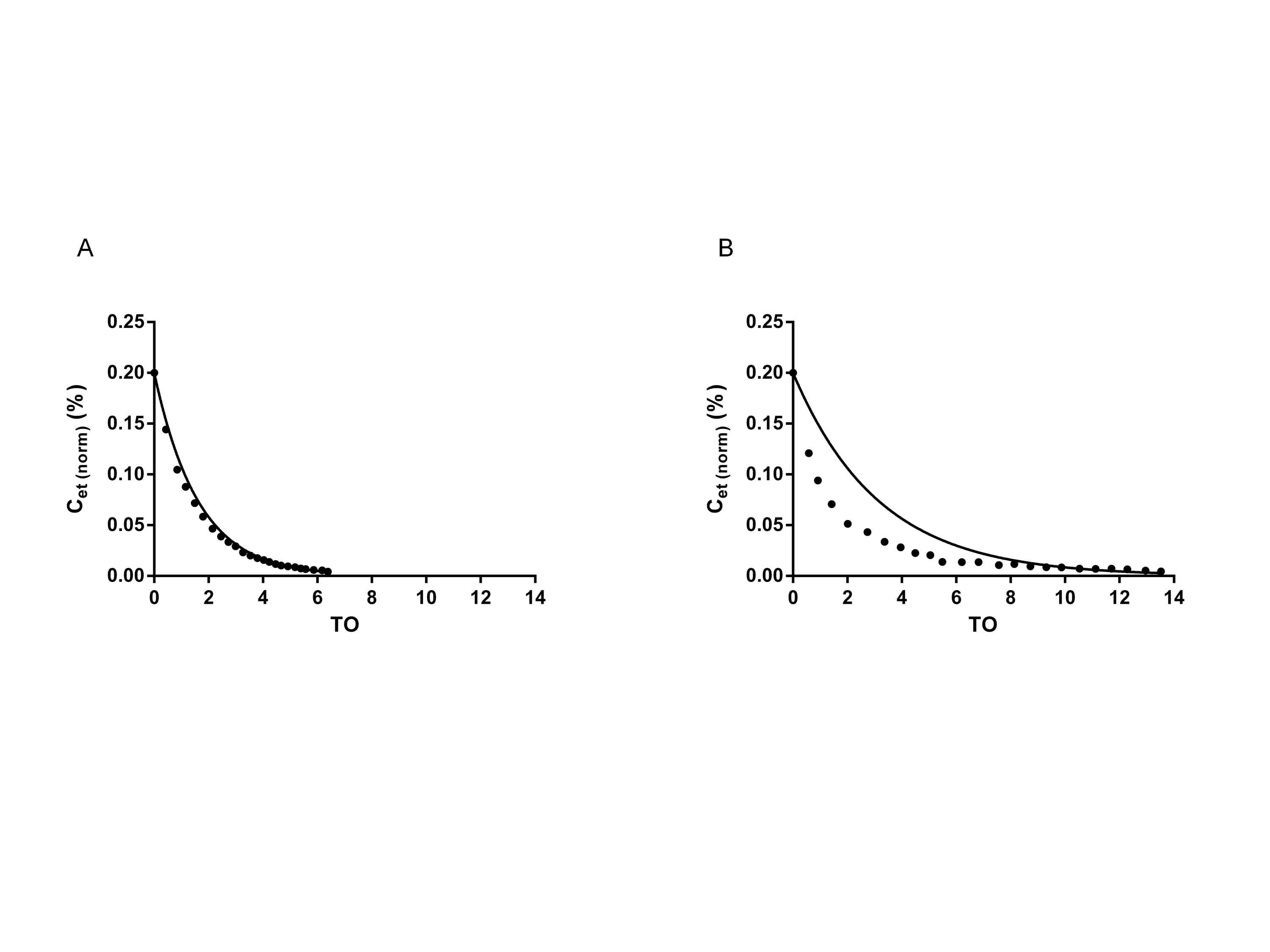

Supplement: Additional file 6: Figure E5 — Washout curves from a healthy subject and a patient with cystic fibrosis fitted to a one-phase exponential decay model. Cet (norm) = normalised end-tidal SF6 concentration; TO = turnover number. Panels A and B show washout curves from a healthy subject and a patient with cystic fibrosis, respectively, fitted to a one-phase exponential decay model. [file 1465-9921-15-59-S6.tiff]

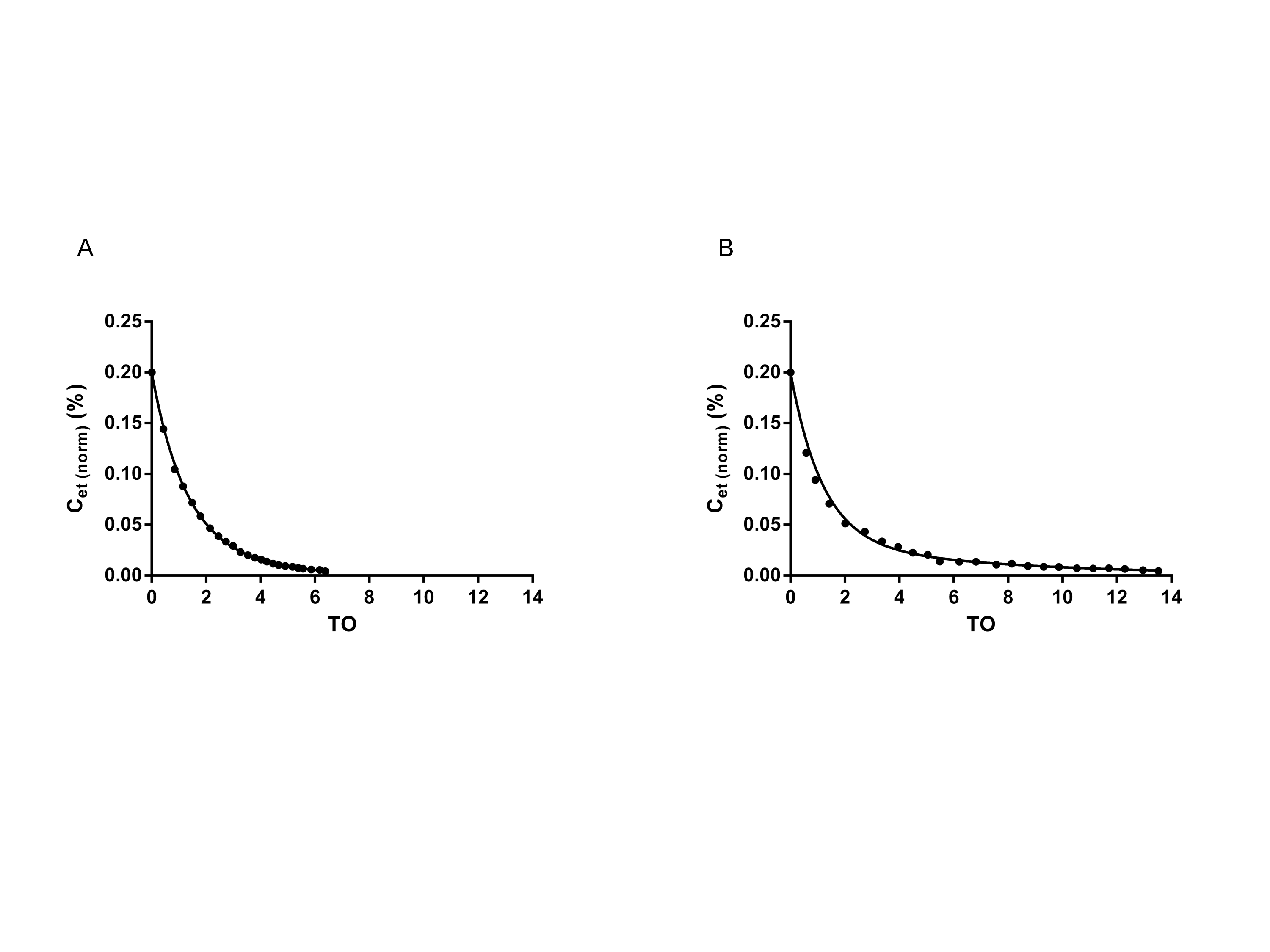

Supplement: Additional file 7: Figure E6 — Washout curves from a healthy subject and a patient with cystic fibrosis fitted to a two-phase exponential decay model. Cet (norm) = normalised end-tidal SF6 concentration; TO = turnover number. Panels A and B show washout curves from a healthy subject and a patient with cystic fibrosis, respectively, fitted to a two-phase exponential decay model. Goodness of fit (R2) = 0.9973 for healthy subject and 0.9775 for cystic fibrosis patient. [file 1465-9921-15-59-S7.tiff]

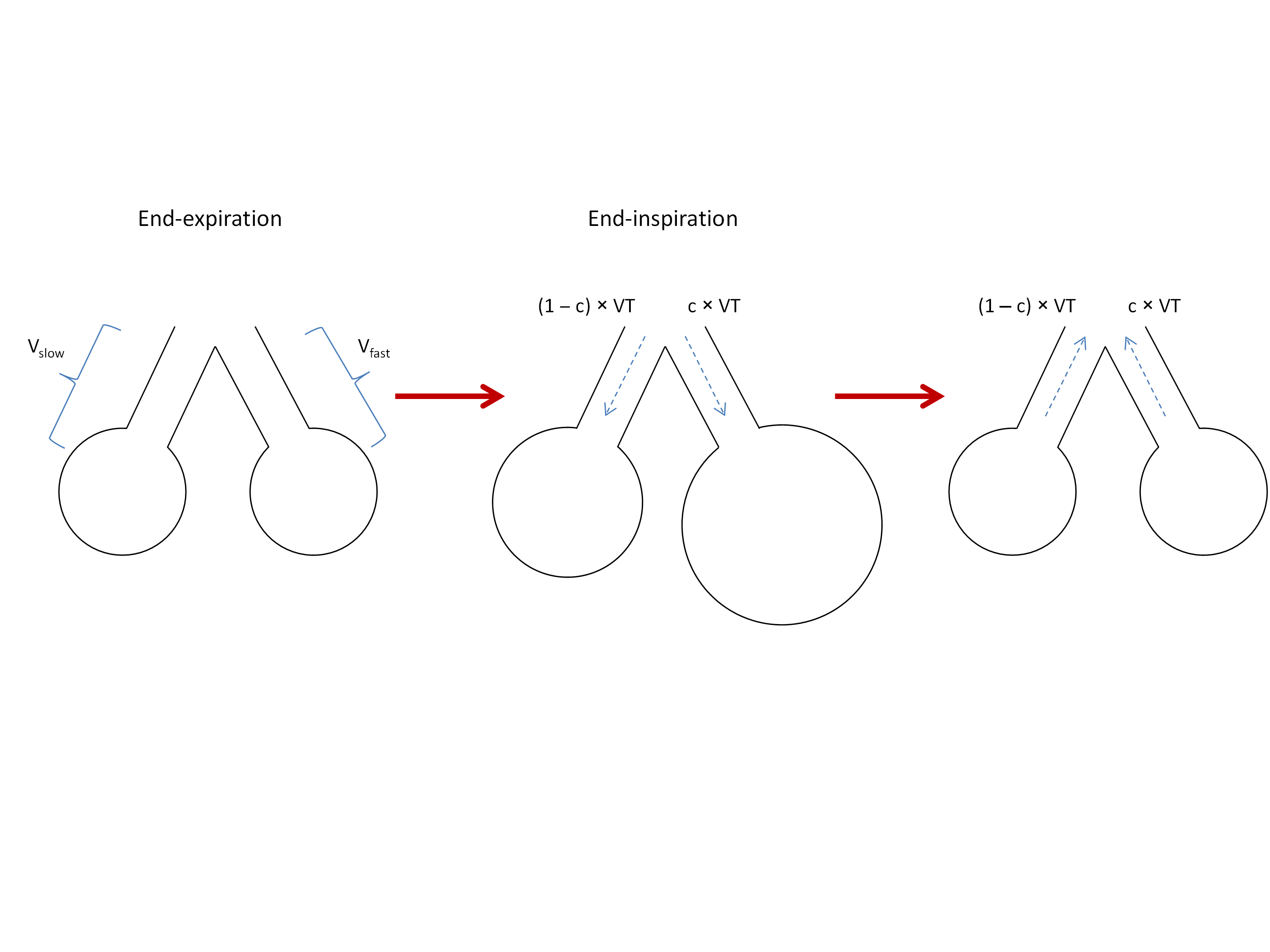

Supplement: Additional file 8: Figure E7 — Two-compartment lung model. Vslow = volume of under-ventilated (slow) lung unit; Vfast = volume of over-ventilated (fast) lung unit; Vt = tidal volume; c = proportion of tidal volume reaching fast lung unit. [file 1465-9921-15-59-S8.tiff]
